# Long-Term Quantitative MRI and Quality-of-Life After Endovascular Therapy in Acute Ischemic Stroke – A MR CLEAN-NO IV Substudy

**DOI:** 10.1007/s12975-026-01497-8

**Published:** 2026-10-13

**Authors:** Praneeta R. Konduri, Dimitrios Karkalousos, Alexandra de Sitter, Manon Kappelhof, Maarten M.H. Lahr, Chi P. Nguyen, Aad van der Lugt, Yvo B. Roos, Jonathan M. Coutinho, Henk A. Marquering, Charles B.L.M. Majoie, Bart J. Emmer, Luka C. Liebrand, Matthan W.A. Caan

**Affiliations:** 1 Department of Biomedical Engineering and Physics, Amsterdam UMC location University of Amsterdam, Amsterdam, The Netherlands; 2 Department of Radiology and Nuclear Medicine, Amsterdam UMC location University of Amsterdam, Amsterdam, The Netherlands; 3 Department of Epidemiology, Health Technology Assessment unit, University of Groningen, University Medical Center Groningen, Groningen, The Netherlands; 4 Department of Radiology, Erasmus MC University Medical Center Rotterdam, Rotterdam, The Netherlands; 5 Department of Neurology, Amsterdam UMC location University of Amsterdam, Amsterdam, The Netherlands

**Keywords:** Brain, Acute ischemic stroke, Long-term follow-up, Quantitative MRI, Quality-of-life

## Abstract

This study aims to explore the long-term evolution of endovascular treatment after acute ischemic stroke (AIS) using quantitative MRI, and its relationship with quality of life (QoL). In this long-term substudy of patients from the MR CLEAN-NO IV trial, patients were included who received 24-hour post-stroke MRI and were functionally independent 90 days after stroke onset. Patients underwent a quantitative MRI exam three years after stroke onset to measure focal volume loss, myelin damage, iron deposition, and atrophy within and around the ischemic lesion seen on 24-hour-MRI, in addition to a QoL assessment. Bayesian statistical analyses were used to assess MRI measures lateralized to the ipsilateral hemisphere and to evaluate the relationship of these measures with baseline and treatment characteristics along with QoL. The Bayes Factor (BF) reflected moderate, strong or very strong evidence (BF = 3.0;20;1.5⋅10^2^ respectively). In the 24 included patients, prominent focal volume loss, iron deposition, ventricular enlargement and myelin damage (BF:2.0⋅10^2^) were observed peripheral to the initial 24-hour DWI lesion at long-term FU MRI. In the basal ganglia nuclei, very strong evidence of focal volume loss (BF > 8.1⋅10^2^) and moderate to very strong evidence of iron deposition (BF: 5.0 to 2.7⋅10^2^) were observed at long-term assessment. Higher systolic blood pressure was associated with reduced long-term ventricular atrophy (BF:5.8). Long-term differences in ipsilateral versus contralateral hippocampal volume difference (BF:8.0) and iron deposition (BF:4.1) were moderately associated with QoL. Long-term subtle yet widespread demyelination and iron deposition were observed, and these changes correlated with long-term recovery.

**Supplementary Information:**

The online version contains supplementary material available at https://doi.org/10.1007/s12975-026-01497-8.

## Introduction

The safety and efficacy of endovascular treatment (EVT) for acute ischemic stroke (AIS) patients has typically been established using clinical outcomes that are reported 90 days after stroke onset [1–4]. Longitudinal data from trials also indicate that the benefit of EVT persists in the long term, up to two years [4–7]. Despite the substantial benefit of EVT, up to half of AIS patients experience cognitive impairment that impacts quality of life (QoL), as measured six months after stroke, and approximately half of patients die within five years of stroke [8–10]. Although it is well-accepted that post-stroke recovery plateaus after three months, the extent of patient recovery can substantially vary due to differences in the degree of acute infarction and treatment characteristics [11].

The onset of stroke triggers a cascade of inflammatory and pathophysiological events that impair the neuro-vascular unit and blood-brain barrier resulting in neuronal death. Recent randomized clinical trials have demonstrated a treatment benefit of EVT compared to medical management alone for patients with extensive ischemic injury [3, 4, 12, 13]. However, favorable functional outcomes remain poorer in patients with more severe strokes [14]. The neurobiology of poorer long-term functional outcome remains understudied.

MRI can measure the impact of ischemic stroke on the brain at both the macro- and microstructural level. E.g., the pathophysiology in the sub-acute period of the first two weeks after stroke onset may be understood by measuring diffusion, perfusion, and lesion volume [15–17]. These studies were constrained to this early phase and did not investigate long-term followup.

Quantitative MRI has been shown to be capable of capturing myelin damage and iron deposition through R_1_ and R_2_^*^ maps, respectively [18]. The temporal progression of subtle myelin damage and iron deposition in R_1_ and R_2_^*^ along with their impact on the functional ability of patients, are well established in neurodegenerative disorders [19, 20]. These studies provide important mechanistic insight but cannot directly inform long‑term post‑stroke tissue changes after thrombectomy. Initial functional and diffusion MRI studies highlighted the complex and heterogenous pathways involved in tissue loss occurring both near and remote from the initial stroke lesion [21, 22], but did not study its microstructural origin.

In this secondary analysis of the MR CLEAN-NO IV trial, the evolution of the stroke lesion was quantified using a comprehensive long-term quantitative MR imaging protocol, targeting a homogenous population of patients with good outcomes (mRS ≤ 3) in which also quality-of-life was assessed, aiming to provide insights into the long-term pathophysiological processes and functional outcome after stroke. The hypothesis is that quantitative MRI reveals persistent microstructural injury and iron accumulation lateralized to the side of the treated occlusion, and that the extent of this chronic brain pathology would be negatively associated with long-term quality of life. By relating these imaging metrics to baseline, treatment, and 24-hour lesion characteristics, we aimed to explore whether EVT influences the long-term evolution of brain damage and functional outcome.

## Methods

### Standard Protocol Approvals, Registrations, and Patient Consents

The present study is a long-term substudy of a selected group of patients from the MR CLEAN-NO IV trial (trial registration: ISRCTN.com, ISRCTN80619088) [23], conducted at Amsterdam UMC. This trial investigated the added benefit of intravenous alteplase prior to intra-arterial thrombectomy in stroke patients with an intracranial occlusion of the anterior circulation. The IRB approved the study as an amendment to the original trial protocol (MEC-2017-368, amendment NL58320.078.17 (v14)) for which all patients had previously agreed to be contacted for possible future studies. Additional informed consent was obtained from all patients or their legal representatives for the long-term study, comprising (1) questionnaires and a phone interview to investigate the long-term clinical and functional outcomes, and (2) an MRI study. These were scheduled within two months of each other, with a median and interquartile range (IQR) of 37 [28 – 38] months after baseline (see Table 1).

**Table 1:** Long-term characteristics of patients. Median values with interquartile ranges are given, unless specified otherwise

Characteristic	Long-term patient group (*n* = 24)
Age (years):	64 [ 51–75 ]
Female sex – no. (%):	8 (33%)
NIHSS-score	15 [ 10–18 ]
Collateral score – score: no.	0:2, 1:6, 2:8, 3:6
ASPECTS – score: no.	6:1, 7:1, 8:8, 9:4,10:10
mRS baseline – score: no. mRS FU – score: no.	0:1, 1:3, 2:17, 3:3 0:2, 1:5, 2:13, 3:4
Systolic blood pressure – mmHg	148 [ 128–163 ]
Treatment with IV alteplase – no. (%)	11 (46%)
eTICI – score: no	0:1, 1:1, 2a:1, 2b:7, 2c:1, 3:11
Intracranial arterial occlusion – no. (%)	Intracranial ICA: 1 (4%) ICA with involvement of the M1 middle cerebral artery segment: 4 (17%) M1 middle cerebral artery segment: 13 (54%) M2 middle cerebral artery segment: 6 (25%)
Long term follow-up inclusion (months)	37 [ 28–38 ]

*NIHSS* NIH Stroke Scale, *ASPECTS* ASPECTS: Alberta Stroke Program Early Computed Tomography Score, *mRS* modified ranking scale, *FU* follow-up, *IV* intravenous, *eTICI* expanded Thrombolysis in Cerebral Infarction, *ICA* internal carotid artery

A small subgroup of patients with good long-term outcomes was targeted. Patients were eligible for the substudy if they had mRS ≤ 3 at 90-day follow-up and the distance from their residence to the coordinating hospital was ≤ 80 km. Other inclusion criteria of the main study are described elsewhere [23]. In the substudy, patients were included during an eight-month period between June 2022 and January 2023.

### Acute and Sub-Acute Imaging

In the main study, Digital Subtraction Angiography (DSA) was performed during thrombectomy. MRI acquisition at 24 h was performed including axial Diffusion-Weighted Imaging (DWI, b = 1000 s/mm^2^), axial Fluid Attenuated Inversion Recovery (FLAIR), Axial T2*-weighted imaging without quantitative R_2_^*^ and Quantitative Susceptibility Mapping, and axial and 3D reconstructed MR angiography (MRA) (3D time-of-flight, TOF).

### Long-Term MRI

#### Acquisition Protocol

In this long-term substudy, MRI data were acquired on a 3T Philips Ingenia (Best, The Netherlands) equipped with a 32-channel head coil. The MR examination consisted of 3D fluid-attenuated T2-weighted imaging, diffusion imaging, 3D quantitative relaxometry, and arterial spin labelling (ASL) perfusion imaging (see Supplemental Table 1).

#### Pre-Processing

The diffusion data were denoised [24], and corrected for Gibbs-ringing [25, 26], distortions [27, 28], motion and eddy currents [29]. Quantitative R_1_ and R_2_^*^ maps were computed from magnetization prepared rapid gradient echo scans at two inversion times (MP2RAGE) and multi-echo gradient echo (ME-GRE) scans in MATLAB (Mathworks, Natick, MA, USA). Quantitative Susceptibility Mapping (QSM) was performed using Laplacian-based phase unwrapping [30]. Arterial Spin Labelling data were processed using ExploreASL [31], including motion correction and partial volume correction.

#### Qualitative Imaging Assessment

Two experienced neuroradiologists (C.M., B.E.) qualitatively scored by comparing the 24-hour imaging to the long-term follow-up imaging for (1) lesion progression, (2) white matter damage, (3) ventricular enlargement, and (4) laminar necrosis. The clinical research form (CRF) used for these readings is provided in Supplementary Table 2. In summary, lesion progression was defined as the presence of the 24-hour stroke lesion as well as focal volume loss on the long-term T1-weighted MRI. White matter damage was defined as new or increased confluent or patchy hyperintensities in the perilesional white matter on the long-term FLAIR MRI, along with susceptibility weighted artefacts defined as hypointensity on long-term T2*-weighted MRI and hyperintensity on R2* and QSM. Ventricular enlargement was defined as ex vacuo dilatation of the lateral ventricle and enlargement on the long-term T1-weighted MRI compared to baseline scan. Cortical laminar necrosis was defined as presence of either linear or laminar hyperintensity confined to the cortical ribbon hyperintensity on the long-term QSM and T1-weighted MRI. Lastly, impact of successful treatment, defined as the degree of reperfusion (expanded Thrombolysis in Cerebral Infarction, eTICI≥2b: 3), was qualitatively assessed on the DSA.

#### Quantifying Lateral MRI Difference Measures

This study aimed to quantify stroke-related iron deposition, myelin damage, volumetric differences, and atrophy three years after stroke onset. To account for intersubject variation, MRI difference measures were computed lateralized to the side of the primary lesion.

Iron deposition in the basal ganglia was quantified in R_2_^*^ maps. Myelin damage proximal to the 24-hour stroke lesion was quantified on R_1_ maps, since R_1_ is known to decrease with decreasing myelin concentration [18]. Ventricular lateral atrophy was computed as the increase in ventricular volume difference from 24-hour to long-term follow-up.

Basal ganglia were semi-automatically segmented on R_1_ maps [32] with manual correction [33]. Segmentations of the lateral ventricles, hippocampus and amygdala were automatically obtained using SynthSeg [34] and visually inspected for accuracy. Normal-appearing white matter (NAWM) proximal to the lesion and in a corresponding region in the contralateral hemisphere was manually annotated in ITK-SNAP as a cubic target volume of 1 cm^3^, excluding voxels residing in non-white matter. Mean anatomical volumes and ΔR_1_- and ΔR_2_^*^-values were computed in MATLAB. Supplemental Fig. 1 illustrates an example segmentation. To assess reliability in NAWM annotation, the Intraclass Correlation Coefficient (ICC) of ΔR_1_ and median distance between ROI positioning of two raters were calculated. To calculate ventricular lateral atrophy at 24 h, lateral ventricles were segmented from the non-diffusion weighted DWI-volume using Synthseg. Tractography of the corticospinal tract was performed using ExploreDTI [35].

#### Structured Interview and Questionnaires

Quality of life (QoL) was assessed through the EQ-5D-5 L questionnaire [36], including five domains (mobility, self-care, usual activity, pain and anxiety, and a combined QoL utility score). Assessments were conducted by a trained research nurse who contacted patients or their caregivers via telephone. Structured interviews took place two or three years post-stroke. During these interviews, respondents reported the patient’s current health status across the five domains, which was subsequently used to calculate the overall utility score using the Dutch tariff [37].

#### Statistical Analysis

Twenty-four-hour DWI lesion volume in substudy participants was compared with that of other trial patients and tested non-parametrically tested using a Mann-Whitney U-test. Bayesian Independent Samples T-tests were performed to assess differences in MRI measures lateralized to the side of the primary lesion, quantifying iron deposition, myelin damage, volumetric differences, and atrophy. Here, the null-hypothesis stated that there was no difference between regions-of-interest corresponding to the stroke lesion and contralateral hemisphere. Furthermore, non-parametric correlation analysis was conducted to quantify the relationship between these MRI lateral difference metrics and EQ-5D-5 L quality of life composite scores and their domain sub-scores. Lastly, the influence of baseline, treatment, and 24-hour lesion characteristics on long-term MRI metrics was tested using qualitative assessment and Bayesian non-parametric correlation analysis. Bayesian statistical analyses were performed in JASP software^7^. The Bayes Factor (BF) was computed, and values of 1.0, 3.0, 20, and 1.5⋅10^2^ were chosen as thresholds for anecdotal, moderate, strong, and very strong evidence, respectively. Kendall’s τ_B_ was computed as the population correlation coefficient. Patients with recurrent lesions on the long-term MRI and those with missing data were excluded from the analysis.

Because this was an observational imaging substudy with a fixed number of eligible patients, no a priori sample size or power calculation was performed; the sample size was determined by feasibility, and the analyses should therefore be interpreted as exploratory, with emphasis on effect sizes and associated Bayes Factors rather than formal hypothesis testing.

## Results

Of the 64 patients from the MR CLEAN-NO IV trial eligible for this substudy, 24 patients were included (Table 1). Intracranial arterial occlusions were predominantly seen in the middle cerebral artery segments (M1: 54%, M2: 25%). Eleven (46%) patients were randomized to the control arm and received IVT plus EVT, while patients in the other arm received EVT only. Of all patients, 19 (79%) achieved successful treatment (eTICI≥2b). In our cohort, the rate of functional independence (mRS ≤ 3) was comparable at 90 days and three years after stroke onset (21 (88%) vs. 20 (83%) patients, BF = 0.3). Stroke recurrence was seen in two patients in the contralateral hemisphere, and these were excluded from quantitative analyses.

### Qualitative Reading

White matter damage within or around the 24-hour lesion was observed in 15 (68%) patients. Focal volume loss and ventricular enlargement were seen in 21 (88%) patients. Susceptibility-weighted artefacts were observed in 17 (77%) patients on QSM, including iron deposition on R_2*_ maps. Hemorrhagic transformation alongside cortical laminar necrosis was observed in 11 (46%) patients with a lesion proximal to the cortex, and two patients had a remaining distal occlusion. White matter damage and focal volume loss were observed on long-term R_1_ and DWI scans in downstream regions corresponding to untreated distal emboli on the post-treatment DSA.

**Fig. 1:**
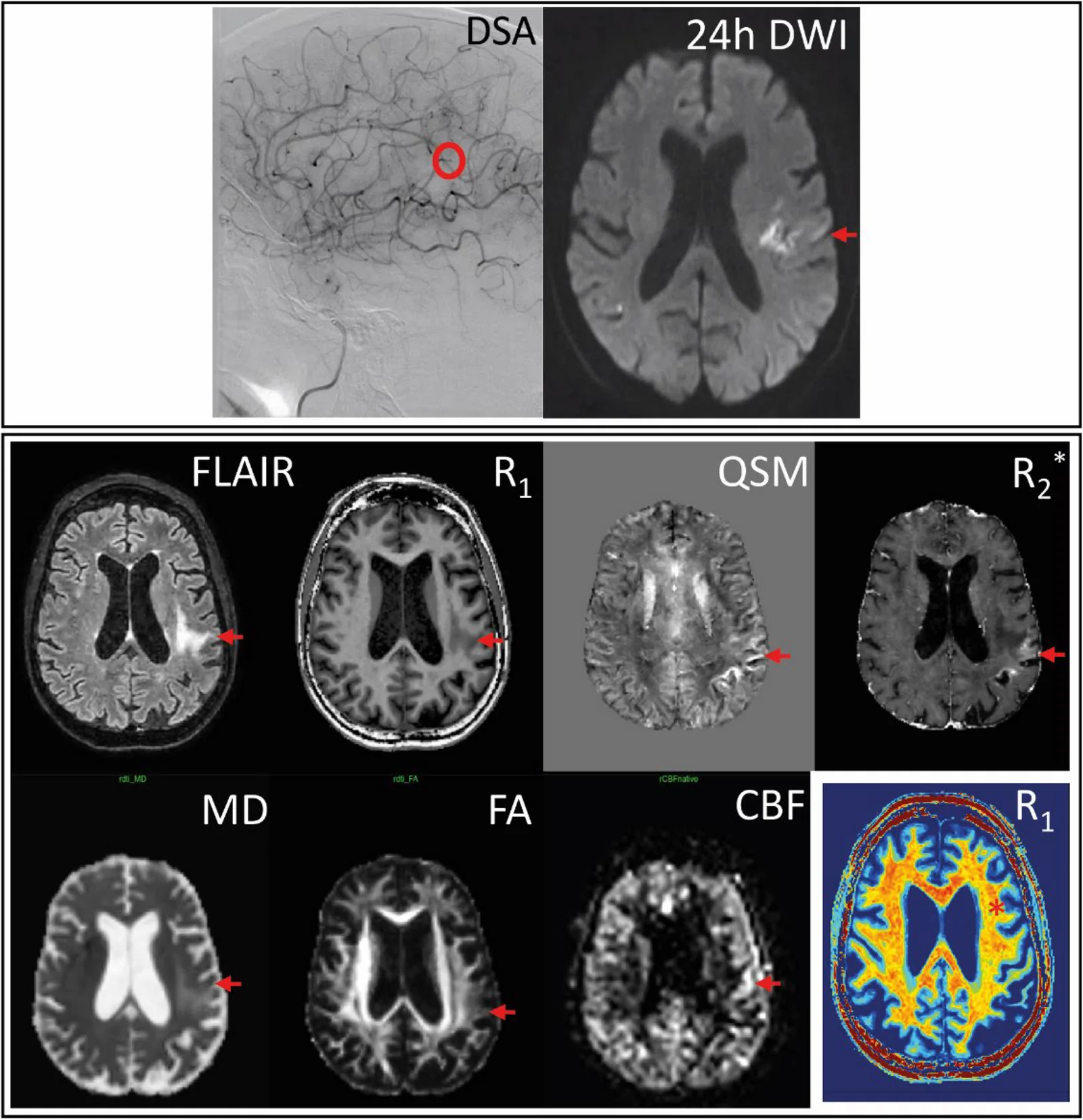
Multimodal imaging of a patient with a left-sided middle cerebral artery (M2-segment) occlusion (ASPECTS = 10) randomized to the intervention arm, successfully treated (eTICI = 3), who was functionally independent (mRS = 2) after 90 days. (Top panel) Thrombus fragmentation was identified on the post-treatment DSA scan with a corresponding lesion on the 24-hour DWI scan. (Bottom panel) Three-year quantitative MRI scans show white matter damage through demyelination (R1, FA), tissue loss and local atrophy around the lesion (FLAIR, CBF, MD), along with cortical laminar necrosis (R2*, QSM). Higher anterior compared to posterior ipsilateral R1 (indicated by an asterisk) is observed

**Fig. 2:**
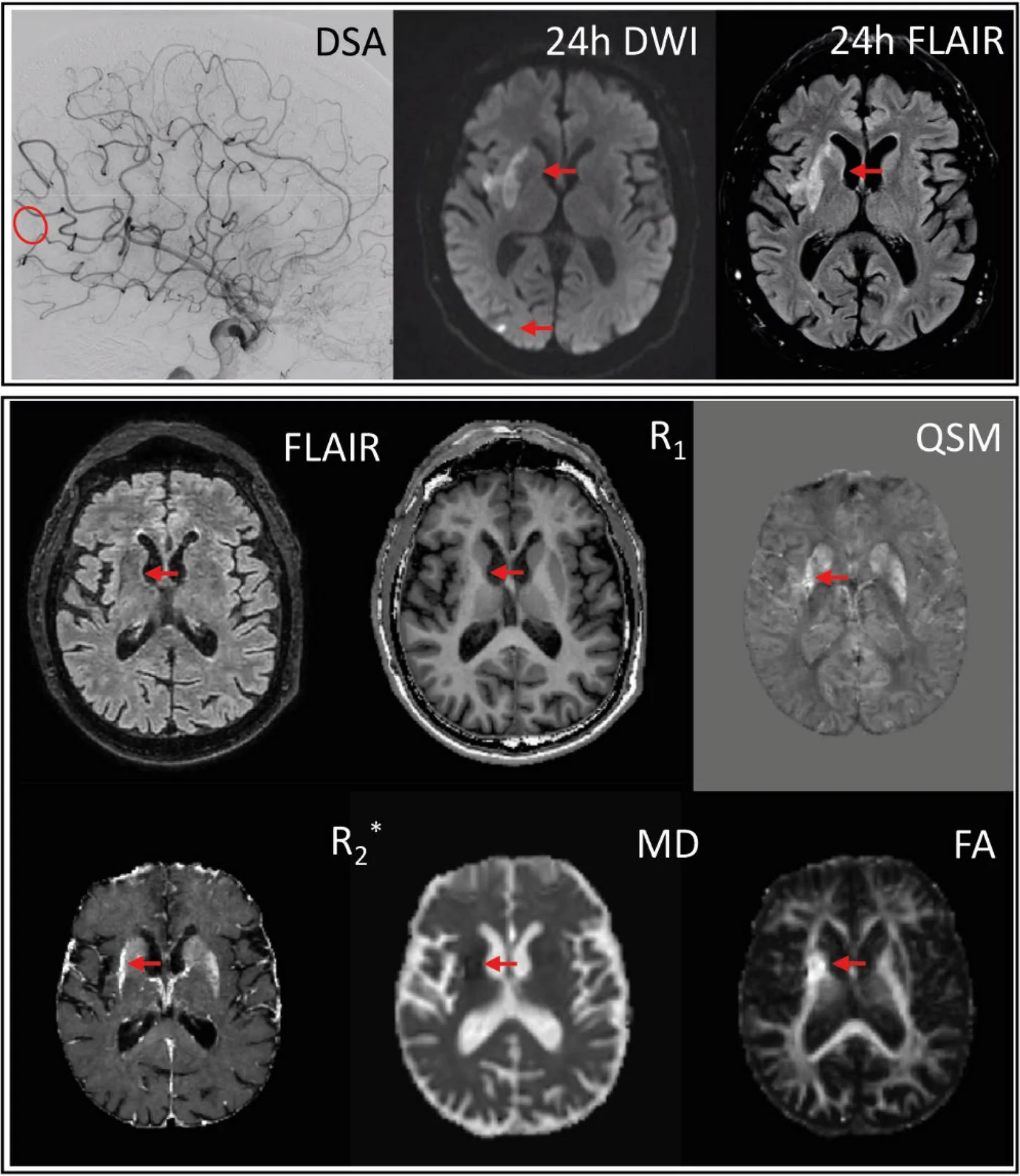
Multimodal imaging of a patient with a right-sided middle cerebral artery (M1-segment) occlusion (ASPECTS = 10) randomized to the intervention arm, successfully treated (eTICI=2c), who was functionally independent (mRS = 1) after 90 days. (Top panel) Thrombus fragmentation was identified on the post-treatment DSA scan with a corresponding lesion on the 24-hour DWI and FLAIR scans. (Bottom panel) Three year quantitative MRI scans show local atrophy around the 24-hour lesion (FLAIR, R1, MD), along with susceptibility artefacts in the M3 region (QSM) indicating hemorrhagic transformation (R2*, FA)

**Fig. 3:**
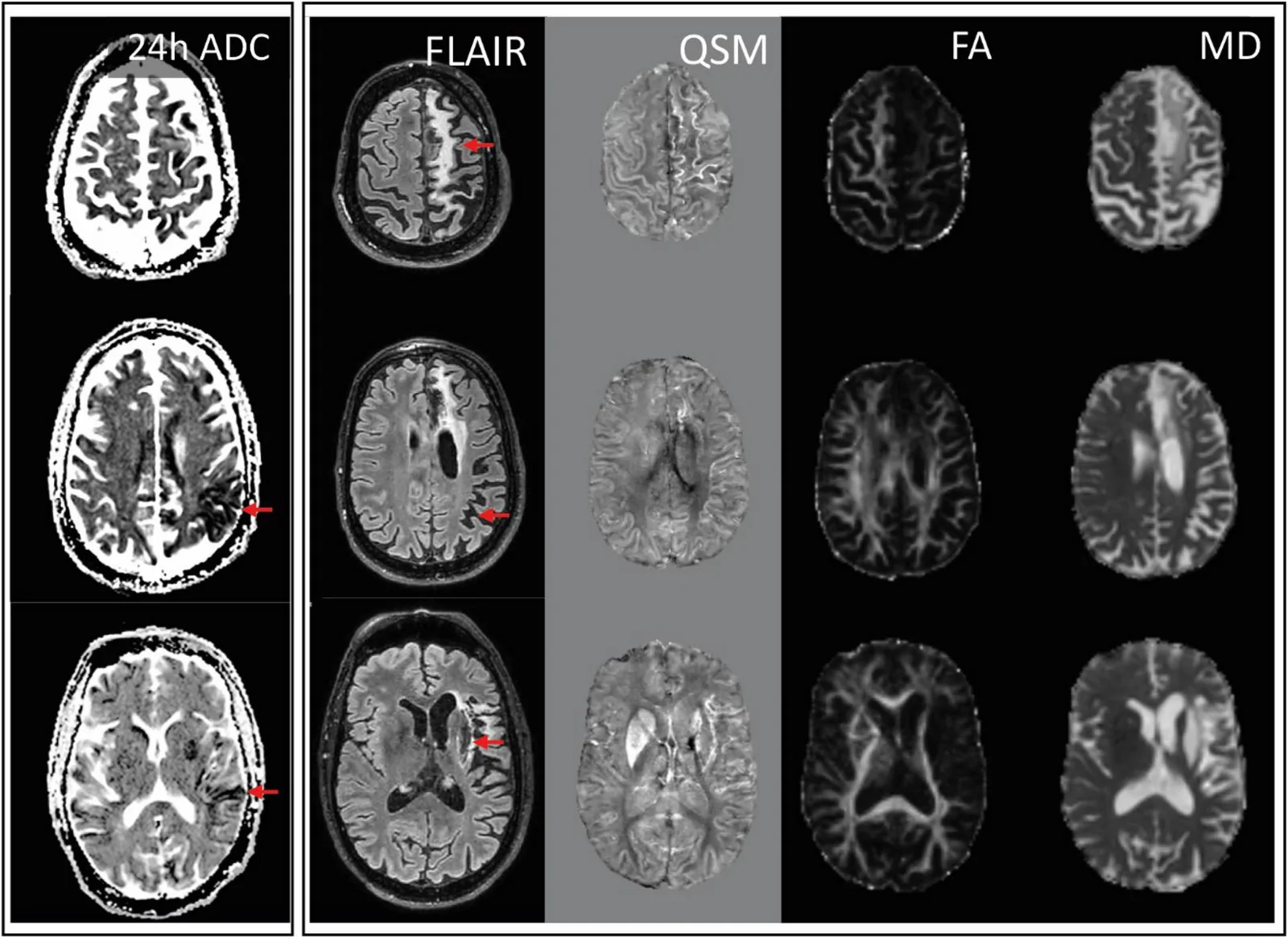
Multimodal imaging of a patient with a left-sided ICA-T occlusion (ASPECTS = 8), and successful reperfusion (eTICI=2b) with slight disability (mRS = 2). Lower apparent diffusion coefficient (ADC) at 24 h led to widespread lesion formation at long-term (right panel)

**Fig. 4:**
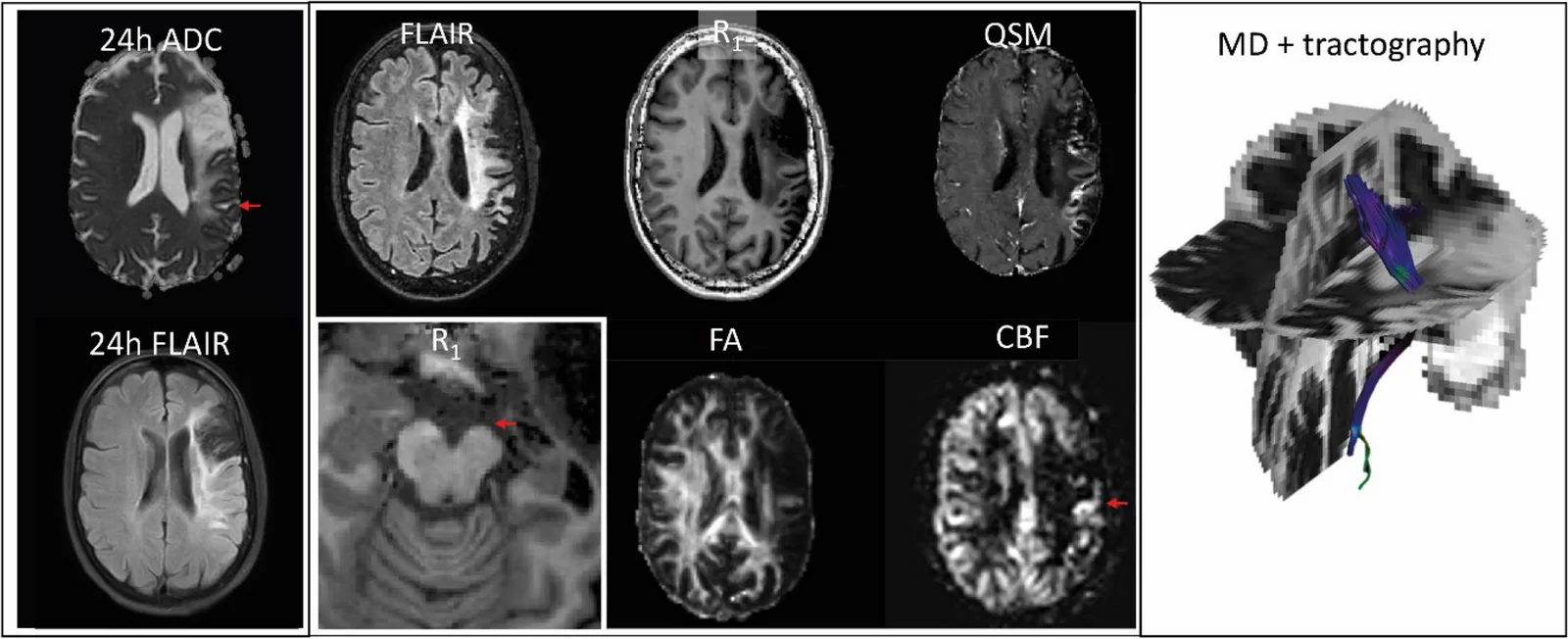
Multimodal imaging at 24 h and long-term follow-up of a patient with left-sided ICA-T occlusion (ASPECTS = 9), successfully treated (eTICI = 3) with moderate disability (mRS = 3) with an old infarct in the insula before and a new infarct in the medial temporal lobe after thrombectomy. (Left panel) Lower apparent diffusion coefficient (ADC) at 24-hours led to lesion formation at long-term. (Middle panel) Transversal panes at the location of the new infarct point to intact perfusion (Cerebral Blood Flow, CBF) along the collateral pathway and are supplemented by an axial slice of R1 at the level of the pons, showing left Wallerian degeneration. (Right panel) Tractography of the corticospinal tract up to the motor cortex

In a representative patient with a left-sided middle cerebral artery occlusion, untreated distal thrombi were identified on the post-treatment DSA scan with a corresponding lesion on the 24-hour DWI scan, which progressed to an extended lesion, lateral ventricular enlargement, and cortical laminar necrosis at long-term assessment (Fig. 1). The R_1_ map indicates lower values proximal to the 24-hour lesion, indicating demyelination. Figure 2 presents a patient with a right-sided MCA occlusion without complete reperfusion. Comparing long-term imaging to the 24-hour stroke lesion, lateral ventricular enlargement was observed, in addition to iron deposition and atrophy in the caudate and lentiform nucleus. Figure 3 illustrates restricted diffusion on 24-hour imaging in a patient with a left-sided internal carotid artery (ICA)-T segment occlusion, and corresponding widespread white matter lesion with lateral ventricular enlargement, cortical laminar necrosis, and atrophy at long-term imaging. Figure 4 depicts a patient with a left-sided ICA-T occlusion who experienced a new infarct after treatment that led to complete asymmetry on FA, lateral ventricular enlargement, atrophy, and cortical laminar necrosis, and ipsilateral Wallerian degeneration at the level of the pons. Tractography showed a successful reconstruction of the entire corticospinal tract, indicative of a spared motor cortex, likely due to good collaterals as seen in the cerebral blood flow (CBF) map.

### Quantitative Analysis

**Fig. 5:**
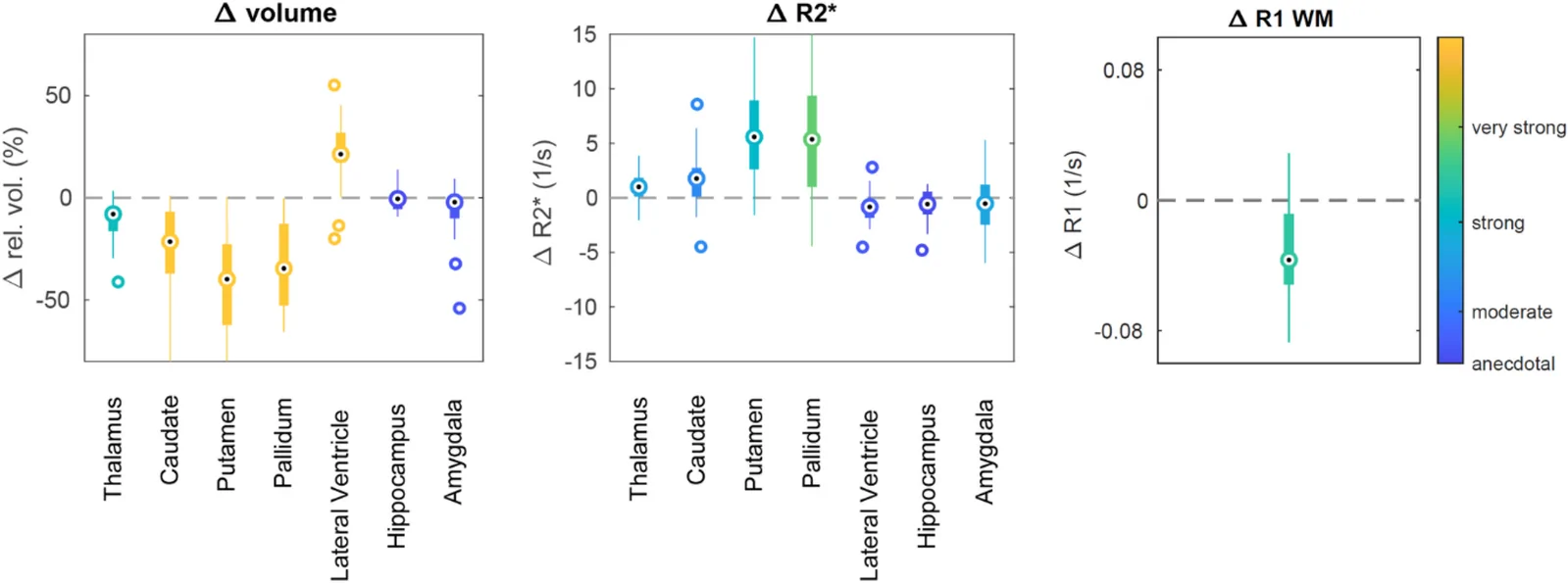
Quantitative volumetric and relaxation parameter differences in ROIs between ipsi- and contralateral hemispheres relative to the primary stroke lesion, colour-coded by the BF indicating the strength of the evidence

The median lesion volume at 24-hour diffusion-weighted imaging in patients enrolled in this substudy of MR CLEAN-NO IV was 14 mL (IQR 8–56). The volume in other patients enrolled in the main trial was 20 mL (IQR 6–75), with no significant difference (*p* > 0.05). In long-term imaging, according to our definition of the Bayes factor, differences ranged from anecdotal to very strong. In NAWM annotations of both raters, strong evidence for a lower R_1_ value in normal-appearing white matter lateralized to the side of primary lesion side was found, indicating myelin damage (Fig. 5). NAWM annotation showed good agreement between raters (ICC = 0.63, median distance 8.8 mm). The lateral differences in R_2_^*^, referring to iron deposition, were largest in the putamen and pallidum, which showed strong-to-very-strong evidence of being higher in the region lateralized to the primary stroke lesion. Although there were no lateral differences in hippocampal and amygdalic volumes, there was strong to very strong evidence for a decreased volume of the thalamus, basal ganglia, and increased volume of the lateral ventricles relative to the corresponding contralateral region of interest.

**Table 2:** Summary of analyses of MRI-metrics in relation to baseline characteristics and long-term Quality of Life. MRI-metrics are reported as lateral differences between ipsi- and contralateral hemispheres, 24 h after treatment (24 h) or at long-term. R_2_^*^ reflects iron deposition. The Bayes Factor (BF) was set to reflect moderate, strong or very strong evidence (BF = 3.0;20;1.5⋅10^2^ respectively). Kendall’s τ_B_ reflects the population correlation coefficient. 95% Confidence Intervals are denoted between brackets

		BF	τ_B_
RRsyst-baseline	ventricular atrophy 24 h	1.9	-0.33 [-0.57; 0.00]
	ventricular atrophy long-term	5.8	-0.40 [-0.62; -0.07]
ASPECTS	thalamus volume	89	0.55 [0.20; 0.74]
Collateral score	thalamus volume	10	0.46 [0.10; 0.67]
proximal occlusion	pallidum volume	1.3.10^2^	0.57 [0.22; 0.76]
	caudate volume	1.0.10^2^	0.56 [0.21; 0.75]
	pallidum R2*	2.4.10^2^	-0.62 [-0.79; -0.25]
	caudate R2*	< 1	0.0 [-0.28; 0.29]
baseline NIHSS	caudate volume	21	-0.48 [-0.68; -0.14]
IVT-administration	Hippocampus R2*	12	-0.46 [-0.67; -0.11]
ventricular atrophy 24 h	ventricular atrophy long-term	4.5	0.40 [0.06; 0.63]
hippocampus volume	Quality of Life	5.9	-0.40 [-0.62; -0.07]
	daily activity	20	0.47 [0.14; 0.68]
hippocampus R2*	Quality of Life	5.0	-0.40 [-0.63; -0.06]
	daily activity	4.0	0.39 [0.05; 0.61]

*RRsyst* systolic blood pressure, *ASPECTS* Alberta Stroke Program Early Computed Tomography Score, *NIHSS* NIH Stroke Scale, *IVT* intravenous thrombolysis

### Association of Baseline, Treatment and 24-Hour Lesion Characteristics with Long-Term MRI Characteristics

**Fig. 6:**
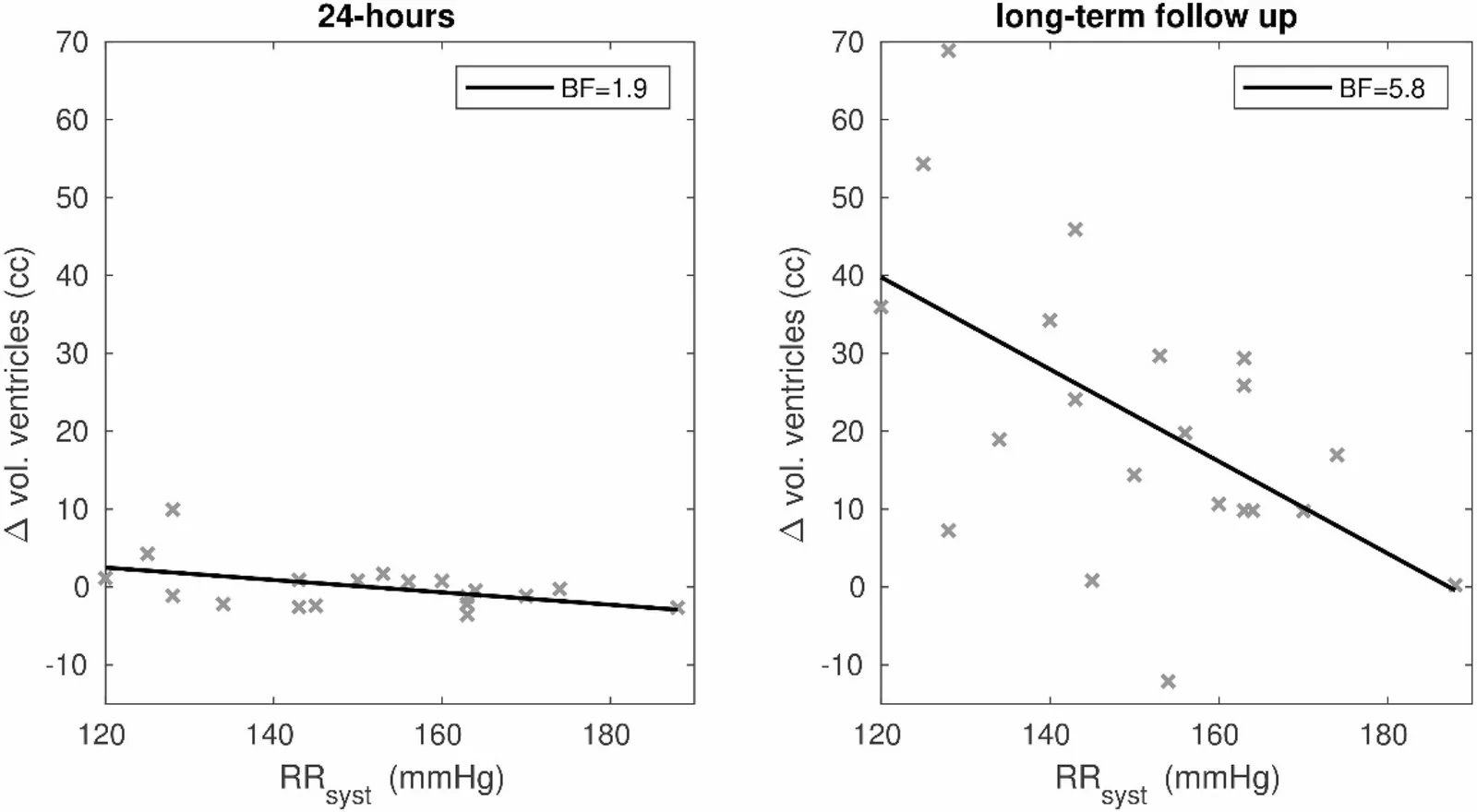
Lateral ventricular atrophy at 24 h (24 h) and long-term, as a function of baseline systolic blood pressure (RR_syst_). The BF (Bayes factor) indicated anecdotal and moderate evidence for an effect at 24 h and long-term

Table 2 summarizes the reported findings. Despite there being no evidence for an association between baseline systolic blood pressure and lateral ventricular atrophy at 24 h (BF = 1.9), higher baseline systolic blood pressure was moderately associated with reduced long-term lateral ventricular atrophy (BF = 5.8, τ_B_=-0.40) (Fig. 6). Lower Alberta Stroke Program Early Computed Tomography Score (ASPECTS) and poorer collaterals were associated with lower ipsilateral thalamic volume compared to the contralateral region-of-interest (BF = 89, τ_B_ = 0.55; BF = 10, τ_B_ = 0.46, respectively). Presence of a proximal occlusion was associated with decreased ipsilateral pallidum (BF = 1.3.10^2^, τ_B_ = 0.57) and caudate (BF = 1.0.10^2^, τ_B_ = 0.56) volumes and higher iron deposition in the pallidum (BF = 2.4.10^2^, τ_B_=-0.62 but not in the caudate (BF < 1). Lower baseline NIH Stroke Scale (NIHSS) was correlated with lower lateral volume differences of the caudate (BF = 21, τ_B_=-0.48). No evidence was found for a relation between successful treatment (eTICI: 3) and any long-term MRI metrics. There was moderate evidence for a lower R2* in the ipsilateral hippocampus after administration of intravenous thrombolysis (IVT) was found (BF = 6.0). Lateral ventricular atrophy at 24 h correlated with atrophy at long-term assessment (BF = 4.5, τ_B_ = 0.40).

### Association of Long-Term MRI with Quality of Life

Higher lateral hippocampal volume difference was moderately associated with a reduction in QoL (BF = 5.9, τ_B_=-0.40). Subsequent analysis showed a strong association between lateral hippocampal volume difference and daily activity scores (BF = 20, τ_B_ = 0.47). Increasing lateralized hippocampal iron deposition was moderately associated with reduced QoL (BF = 5.0, τ_B_=-0.40) and reduced long-term daily activity (BF = 4.0, τ_B_ = 0.39). There was no evidence for an association between other MRI measures and QoL.

## Discussion

This quantitative MRI study of highly selected AIS patients with good functional outcome from the MR CLEAN-NO IV trial demonstrates that subtle yet widespread post-treatment changes persist in the long term, up to three years after stroke onset. Our findings indicate that microstructural myelin damage and iron deposition are apparent on R_1_ and R_2_^*^ maps at long-term assessment, respectively, in addition to macrostructural volume differences. Long-term quantitative MRI metrics were associated with long-term QoL and the ability to perform daily activities.

In normal-appearing white matter proximal to the stroke lesion but outside the original penumbra, small but measurable myelin damage was observed as indicated by lower R_1_ but not FA values lateralized to the side of the primary stroke lesion. This points to subtle but widespread demyelination, while the diffusion anisotropy is comparable. An early diffusion MRI study reported a plateau in FA from one to three years after stroke [38], but did not include R_1_. Future prospective studies may do so, to study causal mechanism of degradation as a primary consequence of stroke in the flow territory, or a process of Wallerian degeneration in nerve fibres distal to the lesion (as observed in a single subject in this study in Fig. 4).

In the basal ganglia, increases in R2* were primarily observed in the pallidum and putamen, which may be attributed to haemorrhage and consequent iron accumulation. Here, a large volumetric difference was observed that co-occurred with iron accumulation. Long-term damage appeared more pronounced in patients with proximal occlusions than in those with distal occlusions, which is consistent with more extensive initial ischemia, but this represents an association and other clinical and biological factors may also contribute. Haemorrhagic transformation is known as a complication of ischemic stroke [39], and involves the basal ganglia [40]. Our study contributes by quantifying the long-term associations between lesion location and cerebral damage.

Quality of life (QoL) was associated with the difference in hippocampal volume and iron deposition lateralized to the side of the primary stroke lesion. Interestingly, no main effect of stroke lesion on hippocampal volume or iron deposition was found. An earlier study into older adults reported that daily activity was positively associated with hippocampal volume [41], supporting our finding. The hippocampus is known to be involved in cognitive decline and memory dysfunction after stroke [42, 43]. The precise causal pathway from baseline characteristics and 24-hour imaging to long-term QoL has yet to be elucidated.

In our cohort, there was moderate evidence for an association between higher systolic blood pressure (SBP) at baseline and a lower difference in lateral ventricular volume, suggesting a potential relationship between SBP and long-term ventricular atrophy. Given the small sample size, observational design, and risk of residual confounding, this association should be interpreted with caution and cannot be taken as evidence that modifying SBP would reduce atrophy or improve prognosis. Previous work referred to a J-shaped or U-shaped association of SBP with a different metric, namely stroke recurrence [44]. There, a detrimental effect on outcomes was observed for SBP above 150 mmHg. Such a cut-off point was absent in our data. Rather, post-hoc inspection showed an increase in lateral ventricular atrophy below 145 mmHg. One possible explanation is that collateral flow could play a role, but this remains speculative and requires confirmation in dedicated studies; it does not justify any specific SBP target in clinical practice.

In a subset of patients, untreated distal emboli and residual occlusions identified on post-treatment DSA were followed by downstream white matter damage, focal volume loss, ventricular enlargement, and cortical laminar necrosis on long-term MRI, despite relatively limited tissue changes on 24-hour imaging. Also, iron deposition and atrophy in the caudate and lentiform nucleus were observed. This pattern, with normal-appearing or only mildly affected tissue at 24 h and pronounced injury three years later, suggests a delayed evolution of secondary neurovascular and neurodegenerative processes rather than an immediate treatment effect. These observations are descriptive and support the plausibility of residual distal thrombus as one contributing factor, but they do not establish that such thrombi are the sole or causal mechanism underlying delayed long-term brain injury.

This study has limitations. Only a small subset of the initial cohort could be included, which was not matched with healthy controls. The lateralized within-subject design controls for subject-level characteristics but provides no such control for the between-subject associations with quality of life. Given the sample size, these associations were not adjusted for key confounders, including age, baseline stroke severity (NIHSS), vascular risk factors, or post-stroke rehabilitation, and should be read as exploratory. Moreover, as a feasibility-constrained observational substudy, no a priori sample size or formal power calculation was performed. Thus, the study may have been underpowered to detect small or moderate associations and findings should hence be interpreted with appropriate caution. Also, our study reports changes three years after the initial insult, which may be caused by other intermediate factors in addition to the initial stroke and EVT procedure. A balanced within subject analysis was therefore designed, comparing ipsi- and contralateral hemisphere metrics, and Bayesian statistical analysis was conducted, reporting the level of evidence for hypotheses in the outcome measures.

This study is biased towards patients with good clinical outcome, who were eligible for EVT, even though not all of them were successfully treated. The results thus cannot be generalized to the broader stroke population. In future work, patients over a broader range of mRS at 90 days post-stroke should be included and assessed at long-term follow-up.

## Conclusion

Patients treated with EVT after AIS in this select cohort showed subtle yet widespread degradation of brain tissue three years after onset, which is associated with long-term QoL. This widespread degradation was observed despite successful reperfusion as seen angiographically in most cases. While unknown factors between the acute and long-term phases could have contributed to the observed associations, quantitative MRI may aid in detecting microstructural changes beyond the apparent stroke lesion and in describing their relationship with functional recovery. Larger prospective multicenter longitudinal studies, ideally with prespecified hypotheses and methods suited for causal inference, are needed before any firm clinical implications can be drawn.

## Supplementary Information

Below is the link to the electronic supplementary material.

Supplementary Material 1 (DOCX 582 KB)

## Data Availability

Data is available upon reasonable request.
