# Supplementary material for: Long-Term Quantitative MRI and Quality-of-Life After Endovascular Therapy in Acute Ischemic Stroke – A MR CLEAN-NO IV Substudy
Source: Transl Stroke Res. 2026 Oct 13;17(5):110. doi: 10.1007/s12975-026-01497-8 (PMC13658416; doi:10.1007/s12975-026-01497-8)
Supplement: Supplementary file 1 [file 12975_2026_1497_MOESM1_ESM.docx]

**Supplementary material**

**Supplemental Table 1. MRI acquisition parameters.** The MP2RAGE scan serves R1-mapping for demyelination, 3D-FLAIR highlights lesions, diffusion MRI renders mean diffusion and fractional anisotropy maps and enables tractography, multi-echo GRE depicts iron deposition in R2* and quantitative susceptibility maps, while pc-ASL yields perfusion maps. Abbreviations: FOV: field-of-view, Acc: acceleration, TR: repetition time, TE: echo time, TI: inversion time, sag/tra: sagittal/transversal, TFE: turbo-field echo, PE: phase-encoding direction, pc-ASL: pseudo-continuous arterial spin labelling, PLD: post-label delay, BG supp: background suppression

| **Sequence type** | **FOV (mm^3^)** | **Acquisition resolution (mm)** | | **Acc. type & factor** | **TR/TE (ms)** | **other** | **Scan time (m:ss)** |
| --- | --- | --- | --- | --- | --- | --- | --- |
| MP2RAGE (3D sag) | 256x244x190 | 1.0 mm iso | C-SENSE 4.6 | | 8.5/ 3.9 | TFE=115 TI=650,2006ms | 4:21 |
| FLAIR (3D sag) | 256x243x190 | 1.15 mm iso  Recon: 0.64 x 0.64 x 0.58 | C-SENSE 12 | | 8000/311 | TFE=186 TI=2400ms | 2:08 |
| Diffusion MRI (tra) | 224x224x97 | 2x2x2  0.2mm slice gap | SENSE 2.4, half-scan 0.75 | | 5500/76 | b=0(6), 1000(34); 3x opp. PE b=0 [s/mm^2^] |  |
| ME-GRE (3D-tra) | 235x176x130 | 1.0 mm iso | C-SENSE 6 | | 38/ 6-30 | ΔTE=6 ms, flyback, FA=17° | 2:48 |
| pc-ASL (tra) | 240x240x133 | 3.0 x 3.0 x 7.0 | SENSE 2.5 | | 4445/15 | PLD: 1800ms; BG supp.; 36 vols.; add. M0-scan | 5:40 |

**Supplemental Figure 1. Regions of Interest** (a) Example automated segmentation of basal ganglia in one R1-map. (b) Manually placed ROIs (yellow: right; blue: left) in bilateral normal appearing white matter (WM) in the R1-map.

**Supplemental Table 2.** Clinical Research Form (CRF) as used in this study.

| CONTRAST IC - MR at **24 *months*** OBSERVER 1 or 2 or 3 \| FU24M_MR STUDY ID: | | | | |
| --- | --- | --- | --- | --- |
| **Study Instance UID:**  **Occlusion side:** | | FULL SI UID: | | |
| **Occlusion location on Baseline CTA:**  Do study ID on CRF and on scan match? □ No □ Yes  Date of scan \|_\|_\| \|_\|_\| \|_\|_\|_\|_\| (dd/mm/yyyy)  Time of scan _____ : _____ (hh:mm) | | | | |
| **24-hour MRI, available?**  Long term Imaging availability:  Quality of the scan  If poor, reason:  Head fully displayed  Vascular malformation or aneurysm visible  Occlusion present (in downstream territory)  New occlusion  If yes, where? | | | **□ No □ Yes**  T2 **□** No **□** Yes  FLAIR **□** No **□** Yes  DWI **□** No **□** Yes  ADC **□** No **□** Yes  T2*GRE **□** No **□** Yes  SWI **□** No **□** Yes  MRA **□** No **□** Yes  dMRI/IVIM **□** No **□** Yes  Other **□** No **□** Yes if yes, other:_____________________  **□** good **□** moderate **□** poor,  Movement □ No □ Yes \| Noise □ No □ Yes \| Artifacts □ No □ Yes other:________________________  **□** No **□** Yes  **□** No **□** Yes  **□** No **□** Yes  **□** No **□** Yes  Infraclinoid ICA **□** No **□** Yes  Supraclinoid ICA **□** No **□** Yes  MCA **□** No **□** Yes if yes, specify MCA segment ______________  Other **□** No **□** Yes if yes, specify ______________ | |
| **Lesion location *** | **White matter damage, present?*** | | **Local atrophy/cortical shrinkage present?** | **Describe tissue damage over time**  Baseline infarct visibility: **□** None **□** Limited **□** Clear  24-hour infarct visibility: **□** None **□** Limited **□** Clear    Susceptibility artefact on long term MR:  **□** No **□** Yes  **Short description of tissue damage:** _____________________________________________________________________________________________________________________________________________________________________________________________________________________________________________________________________________________________________________ |
| **Hemisphere involved: □** Left **□** Right | | | |  |
| □ Caudate nucleus | **□** No **□** Yes | | **□** No **□** Yes |  |
| □ Lentiform nucleus | **□** No **□** Yes | | **□** No **□** Yes |  |
| □ Internal capsule | **□** No **□** Yes | | **□** No **□** Yes |  |
| □ Insular cortex | **□** No **□** Yes | | **□** No **□** Yes |  |
| □ M1 area | **□** No **□** Yes | | **□** No **□** Yes |  |
| □ M2 area | **□** No **□** Yes | | **□** No **□** Yes |  |
| □ M3 area | **□** No **□** Yes | | **□** No **□** Yes |  |
| □ M4 area | **□** No **□** Yes | | **□** No **□** Yes |  |
| □ Other ___________  ___________________ | **□** No **□** Yes  __________________ | | **□** No **□** Yes  __________________ |  |

***Score only baseline lesion, mention in remarks if new lesion present.**

**REMARKS:**

| Name observer: |  |
| --- | --- |
| Signature observer: | ___________________________________ |
|  |  |
| Date of assessment: | \|_\|_\| \|_\|_**\|** \|_\|_\|_\|_\| |
|  | D D M M Y Y Y Y |

_________________________________________

_________________________________________

_________________________________________

_________________________________________

_________________________________________

_________________________________________

_________________________________________

_________________________________________

_________________________________________
